# Ultra-High Energy Density Hybrid Supercapacitors Using MnO_2_/Reduced Graphene Oxide Hybrid Nanoscrolls

**DOI:** 10.3390/nano10102049

**Published:** 2020-10-16

**Authors:** Janardhanan. R. Rani, Ranjith Thangavel, Minjae Kim, Yun Sung Lee, Jae-Hyung Jang

**Affiliations:** 1 School of Electrical Engineering and Computer Science, Gwangju Institute of Science and Technology, Gwangju 61005, Korea; rani@gist.ac.kr (J.R.R.); min7kim9@gm.gist.ac.kr (M.K.); 2 Faculty of Applied Chemical Engineering, Chonnam National University, Gwangju 61186, Korea; ranjith.cecri@gmail.com (R.T.); leeys@chonnam.ac.kr (Y.S.L.); 3 Research Institute for Solar and Sustainable Energies, Gwangju Institute of Science and Technology, Gwangju 61005, Korea

**Keywords:** hybrid supercapacitors, MnO_2_-based supercapacitors, ultra-high energy density, reduced graphene oxide

## Abstract

Manganese oxide (MnO_2_) is a promising material for supercapacitor applications, with a theoretical ultra-high energy density of 308 Wh/kg. However, such ultra-high energy density has not been achieved experimentally in MnO_2_-based supercapacitors because of several practical issues, such as low electrical conductivity of MnO_2_, incomplete utilization of MnO_2_, and dissolution of MnO_2._ The present study investigates the potential of MnO_2_/reduced graphene oxide (rGO) hybrid nanoscroll (GMS) structures as electrode material for overcoming the difficulties and for developing ultra-high-energy storage systems. A hybrid supercapacitor, comprising MnO_2_/rGO nanoscrolls as anode material and activated carbon (AC) as a cathode, is fabricated. The GMS/AC hybrid supercapacitor exhibited enhanced energy density, superior rate performance, and promising Li storage capability that bridged the energy–density gap between conventional Li-ion batteries (LIBs) and supercapacitors. The fabricated GMS/AC hybrid supercapacitor demonstrates an ultra-high lithium discharge capacity of 2040 mAh/g. The GMS/AC cell delivered a maximum energy density of 105.3 Wh/kg and a corresponding power density of 308.1 W/kg. It also delivered an energy density of 42.77 Wh/kg at a power density as high as 30,800 W/kg. Our GMS/AC cell’s energy density values are very high compared with those of other reported values of graphene-based hybrid structures. The GMS structures offer significant potential as an electrode material for energy-storage systems and can also enhance the performance of the other electrode materials for LIBs and hybrid supercapacitors.

## 1. Introduction

A high-density energy storage system is highly in demand for electric vehicles and flexible energy systems requiring a portfolio of various distributed energy resources. Extensive research has been considerably expanded over the last few years to develop innovative green energy-storage devices such as batteries and supercapacitors to cope with highly intermittent demand on electric energy storage [1,2,3,4,5]. Most of the energy-storage devices are based on batteries, which still have low power density and safety issues [6,7]. Supercapacitors have emerged as a promising alternative for Li-ion batteries (LIBs) as they exhibit high power densities, excellent and fast cycling stability, and longevity [1,8,9,10]. Hybrid supercapacitors based on pseudocapacitance (usually based on transition metal oxides) have high energy density than electric double-layer capacitors (EDLCs). Manganese oxide (MnO_2_) has drawn considerable interest as one of the most promising electrode materials for hybrid supercapacitors owing to its ultra-high theoretical energy density (308 Wh/kg for a single electron reaction), elemental abundance in the earth’s crust, and environmental friendliness [11]. However, there are technical challenges to reach the aforesaid theoretical energy density. The low conductivity of MnO_2_ (10^−5^–10^−6^ Scm^−1^) has limited the energy density of MnO_2_-based hybrid capacitors at an unsatisfactory level [12,13,14]. Further, the migration of electrolyte ions into the inner layers is limited by the MnO_2_ surface layer (~400 nm thick). Because of the thick MnO_2_ surface layer, only a part of MnO_2_ can be utilized during the charge/discharge process, which hinders the experimental demonstration of such a high theoretical energy density. The charge–storage mechanism in MnO_2_-based hybrid capacitors is one of the redox reactions involving a change in the Mn oxidation states between 3^+^ and 4^+^, and, for this, they require highly stable MnO_2_. Additionally, poor cycling stability caused by the dissolution of MnO_2_ impedes its commercialization, despite its high theoretical energy density. To overcome the aforementioned issues, such as low energy density and low electrical conductivity, carbonaceous materials are widely used as the conducting platform on which MnO_2_ particles are attached.

Various graphene oxide (GO)/MnO_2_ composites have been utilized as electrode materials for supercapacitors, such as reduced GO(rGO)-carbon nanotubes-based electrodes [15], MnO_2_-deposited graphene/activated carbon film electrodes [16], MnO_2_/MnCO_3_/rGO hybrid nanostructures [17], and hydrothermally derived MnO_2_/GO nanocomposites [18]. These studies focused on growing nanostructured MnO_2_ or synthesizing composites of MnO_2_ and highly conductive rGO. Such strategies primarily enhance electrical conductivity because of the presence of rGO. Regardless of such efforts, their energy density remains low because such composites do not solve the issue of partial utilization of MnO_2_. Thus, the commercialization of MnO_2_-based hybrid supercapacitors is still at stake despite their ultra-high theoretical energy density.

The electrode architecture should be designed so that MnO_2_ particles are anchored on a conducting platform that wraps MnO_2_ to enhance the utilization of MnO_2_, thereby achieving the ultra-high energy density. Scroll-based structures of rGO are an excellent choice for anchoring MnO_2_ particles because (i) rGO has high electrical conductivity, (ii) it provides room for the enhanced utilization of MnO_2_, and (iii) it also solves the problem of the dissolution of MnO_2_. During the formation of MnO_2_/rGO nanoscrolls, the rGO sheets wrap up the MnO_2_ particles anchored on them, enhancing the utilization of MnO_2_. Furthermore, the wrapping prevents the dissolution of MnO_2_. These nanoscrolls can, therefore, overcome issues of low electrical conductivity, material usage, as well as the dissolution of MnO_2_. Thus, ultra-high energy density, power density, and excellent cycling efficiency can be achieved.

A uniform MnO_2_/rGO hybrid nanoscroll structure (GMS) has been synthesized using a simple solution process in the present study. The three-dimensionally connected GMS structure provides a highly conductive path, while the rGO wrap protects MnO_2_ from dissolution. Our GMS/activated carbon (AC) cell delivered a maximum energy density of 105.3 Wh/kg at a specific power density of 308.1 W/kg. It also delivered an energy density of 42.77 Wh/kg at a specific power density of 30,800 W/kg. Our GMS/AC cell’s energy density values are very high compared with those of other reported values of graphene-based hybrid structures [19,20,21]. The cycling performance of the GMS/AC cell suggests that the cell maintained 92% of its initial capacitance even after 10,000 cycles at a current density of 5 A/g, indicating an excellent and stable cycling performance.

## 2. Materials and Methods

### 2.1. Preparation of GO

A modified Hummer’s method via oxidizing graphite powder was used to synthesize GO [22]. The details of the preparation methods are explained in our previously published work [10].

### 2.2. Preparation of MnO_2_ Encapsulated within rGO Nanoscrolls (GMS)

The MnO_2_/rGO nanoscrolls were synthesized by dispersing GO (0.6 g) in 50 mL of water, and 0.15 g of MnO_2_ (purity > 99%, Sigma-Aldrich, Korea) was added to the dispersion and sonicated for 90 min using an ultrasonic reactor. The mixture was then centrifuged at 10,000 rpm for 15 min, followed by filtering. The sonication, centrifugation, and filtering were repeated multiple times, and the solution was dried at 110 °C overnight to form powders. The powder was annealed further at 400 °C in a nitrogen ambient condition for 2 h to remove any impurities. The nitrogen-annealed MnO_2_ encapsulated by rGO nanoscrolls are referred to as GMS and used to fabricate supercapacitors.

### 2.3. Active Material Characterization Measurements

The morphological, structural, and compositional properties of the powders were investigated by using scanning electron microscopy (SEM) and energy dispersive spectroscopy (EDS) mapping (JEOL, JSM-6700F, Tokyo, Japan), high-resolution transmission electron microscopy (HRTEM) (JEM.ARM.200F, Tokyo, Japan), and X-ray photoelectron spectroscopy (XPS) measurements (NEXSA, Thermo Fisher Scientific, Waltham, MA, USA).

### 2.4. Cell Fabrication

Composite anode slurry was synthesized using 75% active materials (GMS), 15% ketzen black (KB), and 10% teflonized acetylene black (TAB) with ethanol, followed by pressing on a 200 mm^2^ nickel steel mesh current collector. Then, the pressed slurry was dried at 160 °C for 4 h in a vacuum oven. Activated carbon (AC) electrodes were also fabricated using a similar method. The active material with a specific mass of 2.5 mg/cm^2^ was used for cell fabrication. The test cells were fabricated using the GMS electrode and AC counter electrode in an argon-filled glovebox. The GMS and AC electrodes were separated by a porous polypropylene film (Celgard 3401, Charlotte, NC, USA) and electrolyte solution of 1 M LiPF_6_ in 1:1 ethylene carbonate (EC)/dimethyl carbonate (DMC). The fabricated cell was labeled as GMS/AC. CR2032 coin cells were fabricated to investigate the hybrid supercapacitor cell’s electrochemical performances in the voltage range of 0−3 V using a battery tester (WBCS 3000, WonATech, Seoul, Korea). The cyclic voltammetry (CV) measurements were conducted at a scan rate ranging from 5 to 50 mV/s. The galvanostatic charge/discharge (GCD) cycling of the cells was also performed in the current density ranging from 0.1 to 10 A/g. The electrochemical impedance spectroscopy (EIS) was conducted in the frequency range of 100 kHz to 100 MHz.

## 3. Results

**Figure 1:**
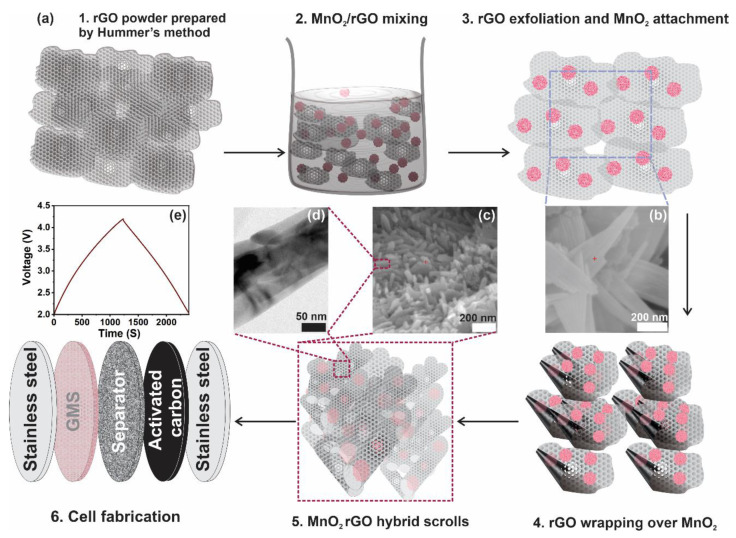
(**a**) (1–6). Schematic showing the formation of nanoscroll structure of GMS active material and the synthesizing procedure of the GMS/AC hybrid supercapacitor, (**b**) scanning electron microscopy (SEM) image of the powder sample showing rGO exfoliation, (**c**) SEM image of the nanoscroll structure of the MnO_2_-encapsulated rGO, (**d**) high-resolution transmission electron microscopy (HRTEM) image of the expanded view of a tubular nanoscroll shown in (**c**), and (**e**) GCD curve of the GMS/AC hybrid supercapacitor at a current density of 0.1 A/g.

Figure 1a (1–6) depicts the schematic of the formation of the nanoscroll structure of GMS active material, illustrating the synthesizing procedure of the GMS/AC hybrid supercapacitor. Figure 1b,c shows the scanning electron microscopy (SEM) images of the powder sample showing rGO flakes after the exfoliation process, and the nanoscroll structure of the MnO_2_-encapsulated rGO active material, respectively. Figure 1d shows the high-resolution transmission electron microscopy (HRTEM) image of the expanded view of a tubular nanoscroll shown in Figure 1c. Figure 1e shows the GCD curve of the GMS/AC hybrid supercapacitor at a current density of 0.1 A/g.

**Figure 2:**
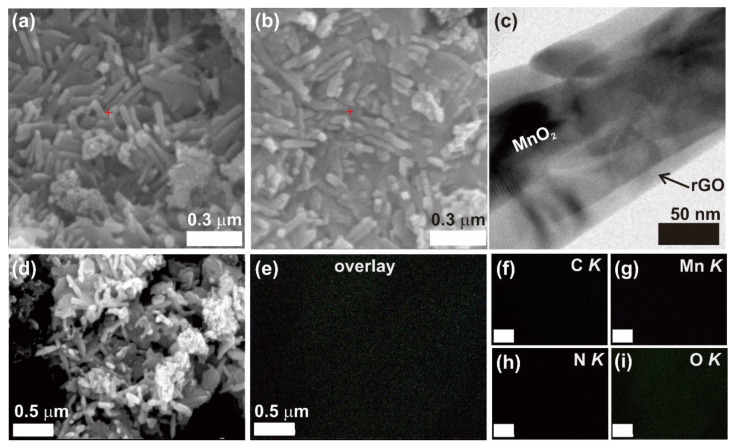
(**a**,**b**). The SEM images and (**c**) TEM image of the synthesized GMS active material confirm the formation of nanoscroll structures; (**d**–**i**) SEM-EDS elemental mapping of the area shown in (**d**).

Figure 2a–c shows the SEM images of the synthesized GMS active material sample, which confirms that the synthesized powder consists of nanoscroll structures. It is apparent from the SEM images that uniform nanoscrolls are formed, and the length of the nanoscrolls is 300 ± 50 nm. Elemental mapping, as shown in Figure 2d–i, was carried out to identify the composition and the distribution of MnO_2_ in the nanoscroll structures. Figure 2d shows the SEM image of the area where the mapping was carried out. The overlay of the C, Mn, N, and O elements is shown in Figure 2e. The elemental mapping of C, Mn, N, and O is shown in Figure 2f–i. The C and Mn mappings confirm that the MnO_2_ particles are well encapsulated in the nanoscrolls. Because the MnO_2_/rGO powders were annealed in a nitrogen (N) ambient condition, traces of N are also observed in the SEM–EDS mapping. The scrolling initiates through the attachment of MnO_2_ particles on to the rGO sheets. The rGO sheets roll up automatically and rapidly to form entangled nanoscroll structures, as shown in Figure 2. More details about the scroll formation can be found in our previous report [23]. The elemental atomic ratio analysis of our rGO/MnO_2_ scroll is shown in Table 1.

**Figure 3:**
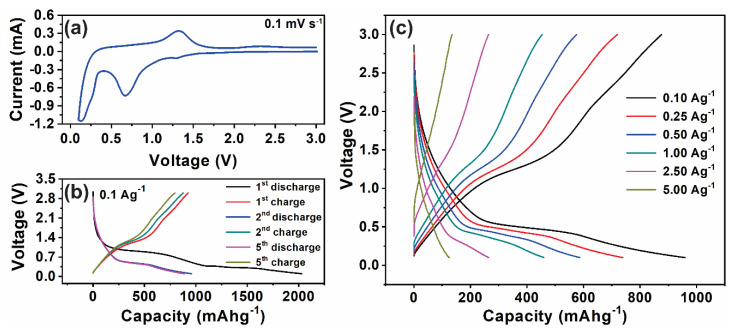
Electrochemical performance of the GMS electrode in a Li half-cell: (**a**) CV analysis of GMS electrode between 0 and 3 V at a scan rate of 0.1 mV/s, (**b**) charge/discharge profiles for the initial stable five cycles, and (**c**) charge/discharge profiles at different current densities.

We have investigated the performance of the GMS/AC cell as a hybrid supercapacitor, and the results are compared to the previously reported works in the following section. Firstly, GMS electrodes were employed as anodes for LIBs, and these electrodes deliver a very high discharging capacity of 2040 mAh/g. The electrochemical activity of the GMS anode material was analyzed using the CV measurements that were performed in the voltage range of 0.01–3.0 V versus Li/Li+, at a scan rate of 0.1 mV/s. The CV curves for the first five cycles in the voltage range of 0.01–3.0 V versus Li/Li+ are shown in Figure 3a. In the first scan, peaks were observed at 0.01, 1.25, and 2.25 V. The peak at 0.01 V, indicates the formation of LiC_6_ through Li intercalation into rGO [24]. The other two distinct oxidation peaks located at 1.25 and 2.25 V indicate that multiple steps are involved in the electrochemical oxidation reaction [25]. The GCD curves (Figure 3b,c) also clearly indicate that two different electrochemical processes occur in the GMS electrode. One is from 0.02 to 1.5 V, and the other is from 1.5 to 3.0 V.

The reaction mechanism is as follows.

The anodic oxidation process of Mn and Li_2_O occurs through the following reaction steps [26]:

Mn + Li_2_O/2Li^+^ + 2e → MnO (1)

MnO + Li_2_O/2Li^+^ + 2e → MnO_2_ (2)

The reduction process of MnO_2_/rGO composite occurs through the following steps:

MnO_2_ + 2Li^+^ + 2e/Li_2_O → MnO (3)

MnO + 2Li^+^ + 2e/Li_2_O → Mn (4)

In the first cycle, the GMS electrode exhibits a very high discharging capacity of 2040 mAh/g. The very high discharge capacity of our battery can be attributed to the specific nanoscroll framework of MnO_2_ wrapped with rGO. More interestingly, the CV curves of the GMS electrode depict the high electrochemical reversibility and stability. The GCD curve measured at a current density of 100 mA/g and in 0.01 to 3.0 V range is shown in Figure 3b. It shows discharge/charge profiles of the GMS electrode at the first, second, and fifth cycles. In the first discharge profile, the GMS electrode exhibits a voltage plateau around 1–0.45 V. The discharge plateau is related to the formation of LiO_2_ and the reduction of MnO_2_ to Mn [26]. Thus, the CV and GCD curves show that the GMS electrode material can perform very well as anodes for LIBs. 

**Figure 4:**
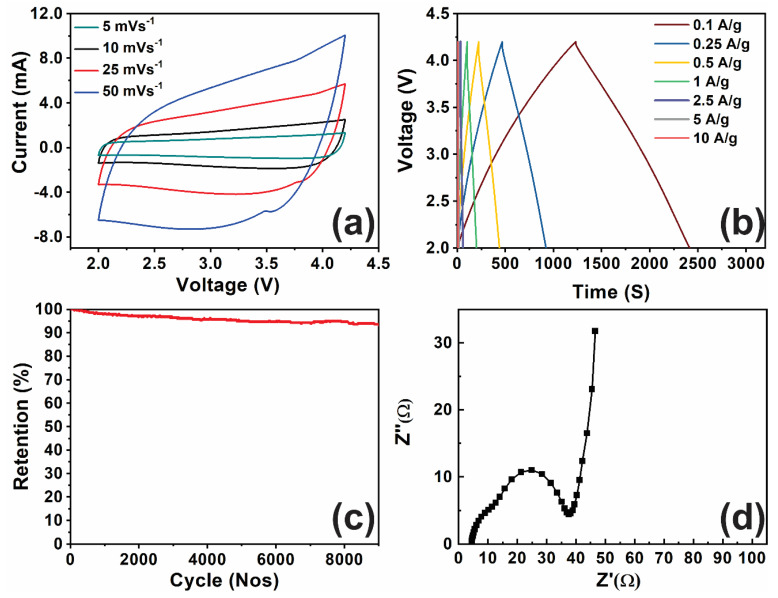
(**a**) CV curves, (**b**) GCD curves of GMS/AC cell, (**c**) the electrochemical stability of the GMS/AC cell, and (**d**) Nyquist plot of the GMS electrode at a frequency range between 100 kHz and 100 MHz with an amplitude of 10 mV in the open circuit condition.

The GMS/AC cell was further investigated for potential use in supercapacitor electrodes and characterized using CV and GCD measurements, as shown in Figure 4a,b, respectively. CV measurements were carried out at a scan rate of 5, 10, 25, and 50 mV/s.

In Figure 4a, the rectangular and symmetric CV curves also suggest the pseudo-capacitive nature of the fabricated electrode. Figure 4b shows the GCD measurements recorded between 2 and 4.25 V and current densities from 0.1 to 10 A/g. From the GCD curve, it is apparent that the charging curve is not symmetric to the discharging curve, further indicating the pseudo-capacitive contribution along with the double-layer contribution. Moreover, the deviation of the GCD curves from an ideal triangular shape indicates that the hybrid supercapacitor exhibits charge/discharge characteristics of a supercapacitor, combined with a battery. Several studies proposed hybrid supercapacitors, which are based on a Faradaic lithium-intercalation cathode/anode (Faradaic) and capacitive anode/cathode (Non-Faradaic) in a non-aqueous electrolyte [27,28]. However, there is an imbalance in the power capability between the two electrodes because Faradaic lithium-intercalation is far more sluggish than a non-Faradaic capacitive reaction. To remedy the sluggish reaction of the intercalation electrode in the hybrid supercapacitor, H. Kim et al. reported a new hybrid supercapacitor with anatase TiO_2_ nanoparticles embedded in reduced graphene oxide as the anode material and activated carbon as a cathode material [29]. They have shown that the CV curve of the hybrid supercapacitors does not follow the curve shape of the symmetric supercapacitor. Instead, it shows a mix of battery and capacitive type performances. They have claimed this by separately measuring the electrochemical performance of anode and cathode using a Li half-cell. In the present study, the CV curves of the rGO/MnO_2_//AC hybrid supercapacitors are different from that of pure EDLC. The deviated rectangular shape and a hump around 3.7 V indicate that the measured capacitance has contributions from the double-layer capacitance and the pseudocapacitance. To claim this, we performed CV analyses of the rGO/MnO_2_ electrode using a Li half-cell and, as shown in Figure 3a, it shows the presence of pseudocapacitance. The CV analysis of the AC electrode using a Li half cell is reported in our previous published work, which shows the presence of EDLC [10]. These results confirm the nature of capacitive and redox reaction mechanisms in the hybrid supercapacitors.

The charge storage mechanism of MnO_2_ has been reported previously through the following reaction mechanism [30]:

MnO_2_ + Li^+^ + e^−^ ↔ MnOOLi (5)

The storage mechanism involved a redox reaction between the III and IV oxidation states of Mn.

During the charging operation, lithium ions from the electrolyte react with MnO_2_ in the GMS anode, providing the pseudocapacitance effect (battery type). The simultaneous adsorption of anions (PF^6−^) from the electrolyte over the positive AC electrode provides the double-layer capacitance effect (supercapacitor type).

However, during discharging, MnO_2_ is regenerated, with simultaneous anionic desorption from the AC. The capacitive response from the hybrid capacitor results from the combinatory behavior of the electric double-layer charge storage at the AC cathode and the redox reactions at the GMS anode.

The mass loading of the active material was 2.5 mg/cm^2^. The mass balance between the electrodes significantly affects the energy storage of hybrid supercapacitors. Because the electrodes are different, the applied current is divided depending on the capacitance of the electrodes. Therefore, to balance the current flow, the mass loading was adjusted based on each electrode’s electrochemical performance, carried out in a single electrode configuration (Li/MnO_2_/rGO and Li/AC, where metallic lithium acts as a counter and reference electrode).

Thus, the GMS/AC cell performs very well as a hybrid supercapacitor, which is the combination of a supercapacitor and the battery.

The gravimetric capacitance is calculated using the following equation:

C = i/slope, where i is the applied current, and the slope is equal to dV/Δt.

The details of the equation can be obtained from previous reports [31,32]

Thus,

(6) $$C_{gra}=4\frac{i\Delta t}{\mathrm{mdV}}$$

where i is the applied current, m is the mass of the active material, including both the anode and cathode in the cell, and Δt and dV are the discharge time and potential window, respectively.

The gravimetric specific capacitance of the cell is found to be 223.2 F/g.

The gravimetric energy- and power-density values can be calculated from the following equations [31,32].

Gravimetric energy density:

(7) $$Egra=\frac{1}{m}\int_{0}^{t_{d}}iVdt$$

Gravimetric power density:

(8) $$P_{gra}=\frac{E_{gra}}{\Delta t}$$

where *m* is the mass of the active material, including both the anode and cathode in the cell, *i* is the discharge current, *V* is the potential, and *t_d_* is the discharge time

The GMS/AC cell delivered a maximum energy density of 105.3 Wh/kg and a corresponding power density of 308.1 W/kg. It also delivered an energy density of 42.77 Wh/kg at a power density of 30,800 W/kg. The cycling performance of the GMS/AC cell (Figure 4c) suggests that the cell maintained 92% of its initial capacitance even after 10,000 cycles at a current density of 5 A/g, indicating excellent and stable cycling performance. We have already published the performance of rGO in our previous works, and the rGO/AC cell delivered a maximum energy density of only 70 Wh/kg [33].

EIS studies were conducted for the GMS/AC cell at a frequency range between 100 kHz and 100 MHz and with an amplitude of 10 mV in open circuit conditions, as presented in Figure 4d. The Nyquist plot consists of semicircles and a sloping line in the high-to-medium frequency region and the low-frequency region. In the low-frequency region, the slope of the line represents the diffusion-controlled process. The slope of the line at low frequency provides the diffusion rate of ions. The higher the slope, the faster the diffusion. The semicircle intercept in the high-frequency region with the real impedance axis (Z’) represents the equivalent series resistance (ESR). The calculated ESR of the GMS/AC cell was 4 Ω. It is worth mentioning that the ESR of 4 Ω of our GMS cell is lower than that of other reported values for rGO-based electrodes such as the MnO_2_–Au nanofiber-based supercapacitor electrode (22.52 Ω) [34], the carbon nanotube–MnO_2_ electrode-based supercapacitor (21.8 Ω) [20], the carbon fiber–MnO_2_ electrode-based supercapacitor (35.58 Ω) [19], and the Na-doped MnO_2_ nanosheets-carbon nanotube fiber electrode-based supercapacitor (8.7 Ω) [35]. The charge–transfer resistance (R_ct_) resulting from the diffusion of electrons towards the electrode materials can be estimated from the semicircle diameter. The shorter value of the semicircle diameter suggests that the R_ct_ of the GMS/AC cell is 35 Ω. This low Rct value is because of GMS’s nanoscroll structure, which facilitates the electrochemical transport of the electrolyte ions into the MnO_2_ and provides a connected network through the scroll structures, resulting in the rapid transport of Li ions.

**Figure 5:**
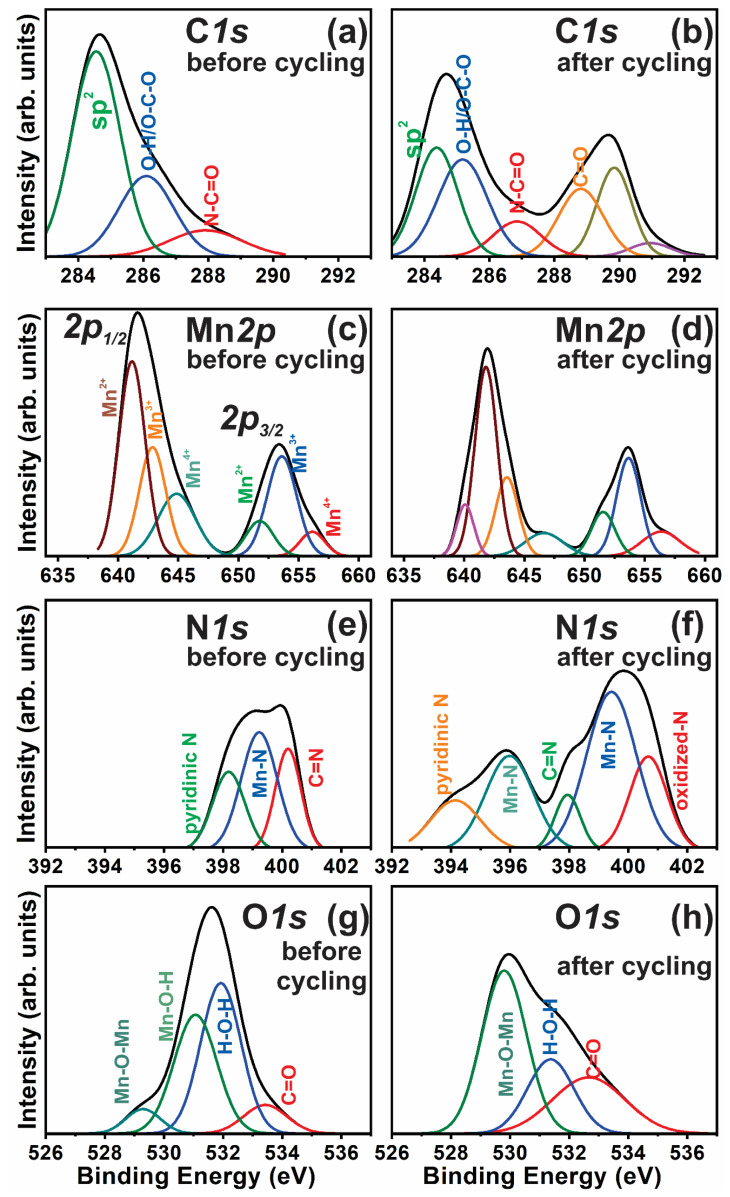
The XPS spectra and corresponding fitted peaks of (**a**,**b**) C*1s*, (**c**,**d**) Mn*2p*, (**e**,**f**) N*1s*, and (**g**,**h**) O*1s* before and after cycling.

Figure 5 shows the fitted XPS spectra (C1s, Mn2p, N1s, and *O*2p) of the GMS electrode material before and after cycling. The capacitance variation after 10,000 cycles and the electrochemical reversibility of GMS electrode can be analyzed by investigating the oxidation state of the constituent elements in GMS, before and after the cycling process.

To find out the oxidation states of the constituent elements in GMS, C1s, Mn2p, N1s, and O2p, the XPS of GMS has been recorded. The fitted peaks and assignments of peaks are tabulated in Table 2. The C*1s* XPS spectra of the GMS electrode before cycling, shown in Figure 5a, suggests the presence of various functional groups such as sp^2^ carbon, O–C–O, and N–C=O. The peak at 284.4 eV corresponds to sp2 carbon, while 287.9 eV corresponds to N-C=O.

The Mn*2p* XPS spectra of the GMS electrode before cycling (Figure 5c) consists of Mn *2p*_3/2_ and Mn *2p*_1/2,_ centered around 641.5 and 653.4 eV, respectively, with an energy separation of 11.9 eV. The energy separation of 11.9 eV is in good agreement with the energy difference observed for MnO_2_. The C–N and Mn–N bonds are observed from N*1s* XPS spectra (Figure 5e). O*2p* XPS shows (Figure 5g) the binding energy (BE) peak positions corresponding to the Mn–O–Mn, Mn–O –H, and C=O bonds. Thus, the peaks corresponding to 529.7 eV (Mn-O-Mn), 531.3 (Mn-O-H), 532.7 (H-O-H) are observed from the O1s spectra for GMS before the cycling process. For GMS after cycling, the sample’s O1s spectrum has been deconvoluted into individual peaks corresponding to 529.2 eV (Mn-O-Mn), 531.1 eV (Mn-O-H), 531.8 eV (H-O-H), and 533.4 eV (C=O).

To investigate the effect of cycling in the GMS electrode, the XPS spectra of the GMS samples after cycling were also recorded and are shown in Figure 5b,d,f,h. After cycling, significant changes are observed in C*1s* and N*1s* spectra. The peak at 285.2 eV corresponds to C-N bonding. N–C=O bonds observed in N*1s* spectra before the cycling break and form only C=O bonds. Oxidation of the carbon is significant due to the effect of the cycling of the GMS sample. An additional shoulder peak at 646.4 eV appears in the Mn*2p* XPS spectra of the GMS sample after cycling, whereas it was absent before the cycling. This shoulder peak indicates the presence of Mn^3+^ ions. The XPS analyses indicate that humid conditions alter the Mn valence states because of MnO_2_ and water vapor interaction. The XPS results show that the formation of C=O, Mn–OH, and breakage of Mn–N bonding after the cycling results in the cell fabricated performance degradation using GMS.

**Figure 6:**
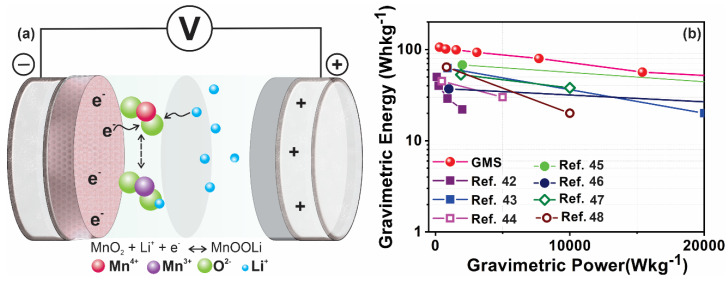
(**a**) Schematic of the charge/discharge mechanism, and (**b**) Ragone plots of the proposed GMS/AC cell in comparison with reported values [42,43,44,45,46,47,48].

Figure 6a shows the schematic of the charge/discharge mechanism. The energy density values of our GMS/AC cell are much higher than those of the other previously reported graphene hybrid structures [42,43,44,45,46,47,48]. The Ragone plots of the recently reported graphene-based hybrid supercapacitors are compared to those of the proposed GMS/AC cell and are shown in Figure 6b. The gravimetric energy density of the GMS/AC cell was higher than those of other very recently reported devices, such as the carbon nanotube–MnO_2_ supercapacitor (42 Wh/kg) [20], the carbon fiber–MnO_2_ supercapacitor (49.4 Wh/kg) [19], the MnO_2_@CNTs/Ni network-based symmetrical supercapacitor (94.4 Wh/kg) [21], the graphene/MnO_2_ nanoparticle hydrogel-based asymmetric supercapacitor (21.2 Wh/kg) [49], the conjugated indole-based macromolecule (30 Wh/kg) [50], the graphene/RuO_2_ hybrid capacitor (20.1 Wh/kg) [51], rGO/mixed-valence MnO_2_ composite (50 Wh/kg) [52], GS/Ni(OH)_2_ nanoplates (53 Wh/kg) [46], thermally reduced GO supercapacitors (62.5 Wh/kg) [43], porous Ni_3_S_2_/CoNi_2_S_4_ three-dimensional-network structure (62.2 Wh/kg) [53], asymmetric supercapacitors based on ionic liquid complex intercalated rGO (67.8 Wh/kg) [48], highly graphitic carbon–tipped MnO_2_/mesoporous carbon/MnO_2_ hybrid nanowires (37 Wh/kg) [45], ionic-liquid-assisted Cu_2_O nanoparticles/multi-walled carbon nanotube nanocomposite (64.2 Wh/kg) [47], and mixed-valence sulfur-doped V_6_O_13−x_(45 Wh/kg) [44]. Thus, the excellent performance of the GMS/AC cell will find suitable applications that demand high energy density storage devices.

## 4. Conclusions

We designed and fabricated a hybrid supercapacitor, utilizing MnO_2_/rGO nanoscrolls as an anode. The GMS/AC hybrid supercapacitor exhibited enhanced energy density, superior rate performance, and promising Li storage capability that can bridge the energy density gap between the conventional LIBs and supercapacitors. The lithium storage specific capacity was 2040 mAh/g, one of the highest values achieved by the hybrid supercapacitor systems based on intercalation compounds. At a voltage range from 0 to 3.0 V, the GMS/AC cell delivered a maximum energy density of 105.3 Wh/kg and a corresponding power density of 308.1 W/kg. It also delivered an energy density of 42.77 Wh/kg at a power density of 30,800 W/kg. The excellent and stable cycling performance of the GMS/AC cell was also demonstrated. It maintained 92% of its initial capacitance even after 10,000 cycles at a current density of 5 A/g. XPS analysis confirmed that excellent cycling stability is achieved because the rGO wrapping prevents the dissolution of MnO_2_. The developed MnO_2_/rGO hybrid nanoscroll structure offers excellent potential as an electrode material for energy storage systems.

## Figures and Tables

**Table 1:** The elemental atomic ratio analysis of the rGO/MnO_2_ scrolls.

Element	Atomic Percent (%)
C	30.58
N	1.32
O	40.03
Mn	28.07

**Table 2:** The fitted peaks and corresponding assignments of the GMS electrode before and after cycling.

Sample	B.E (eV) C1s	Assignment	Ref.	B.E(eV) Mn*2p*_3/2_	B.E (eV) Mn*2p*_1/2_	Assignment	Ref	B.E (eV)	Assignment	Ref.	B.E (eV)	Assignment	Ref
GMS before cycling	284.4 286.2 287.9	sp^2^ carbon O-H/O-C-O N-C=O	[36]	656.1 653.5 651.8	644.9 642.8 641.1	Mn^4+^ Mn^3+^ Mn^2+^	[37]	398.2 399.2 400.2	Pyridinicnitrogen Mn-N34 C=N	[38]	529.7 531.3 532.7	Mn–O–Mn Mn–O–H H–O–H	[39]
GMS after cycling	284.4	Sp2 carbon	[36]	656.5			[40]	394.1			529.2	Mn–O–Mn	[39]
	285.2 286.8	C-N O-H/O-C-O	[36]	653.6 651.5	646.6	trivalent Mn ions of MnOOH		396 397.9	Pyridinic nitrogen	[38]	531.0 531.8	Mn–O-H H-O-H	
	288.8 289.8	C=O	[36]		643.6 641.8 640.08	Mn4 + Mn3 + Mn2 +	[37]	399.4 400.6	Mn-N Oxydised itrogen	[41]	533.4	C=O	[36]
